# Significant reduction in creep life of P91 steam pipe elbow caused by an aberrant microstructure after short-term service

**DOI:** 10.1038/s41598-024-55557-w

**Published:** 2024-03-03

**Authors:** Hongyu Zhou, Jian Li, Jie Liu, Peichen Yu, Xinyang Liu, Zhiyang Fan, Anqing Hu, Yinsheng He

**Affiliations:** 1 National Center for Materials Service Safety, University of Science and Technology Beijing, Beijing, 100083 China; 2 Jiangsu Xihu Special Steel Group Co., Ltd, Taizhou, 225721 Jiangsu China; 3 Aerospace Science & Industry Defense Technology Research and Test Center, Beijing, 100854 China; 4 Key Laboratory of Special Equipment Safety and Energysaving for State Market Regulation, China Special Equipment Inspection & Research Institute, Beijing, 100029 China; 5 China Railway Engineering Group Tunneling Equipment Manufacturing Co., Ltd, Xinxiang, 453000 Henan China

**Keywords:** Structural materials, Energy infrastructure

## Abstract

P91 steel is an important steam pipe for ultra-supercritical power plants due to its excellent creep strength, which generally has a design life of 100,000 h. Here, we found a significant aberrant decrease in the creep rupture life of a main steam pipe elbow after only 20,000 h of service. The microstructure in the aberrant piece exhibited a decomposition of martensitic lath into blocky ferrite due to recrystallization and accumulation of M_23_C_6_ as well as formation of the Laves phase along the prior austenitic grain boundaries, resulting in the decrease of hardness that no long meet ASME standard requirement. The creep testing of the P91 piece at 550–600 °C and 85–140 MPa shows that the influence of temperature on the cavity formation and cracking is greater than that of the applied stress. The rupture life is nearly two orders of magnitude shorter than the normal P91, attributing to the creep damage of the subgrain growth, M_23_C_6_ and Laves phase coarsening (aggregation approaching 3.4 μm). The residual life of the aberrant piece was evaluated to be 53,353 h based on the Larson–Miller parameter, which is much shorter than the design life, suggesting the safety operation of the elbow area should be paid more attention during the afterward service periods. P91 steel, main steam pipe elbow, aberrant microstructure, service degradation, creep life prediction

## Introduction

Due to its excellent high-temperature strength, corrosion resistance and heat-transfer properties, the P91 steel has been widely used for power generation components such as steam generators, intermediate heat exchangers, and coal-fired power plants ^1–3^. However, the creep damage of P91 steel in long-term service is one of the prime causes of structural aging and degradation, which can lead to premature failure accidents^4,5^. Plant operators normally expect a service life of at least 100,000 h, and normal tempered martensitic steels such as P91 with complex microstructures meet this qualification^6^. However, the bending process of the main steam pipe involving a large amount of deformation can produce an aberrant microstructure of the heat-resistant steel, which may lead to significant changes in creep characteristics^7^.

In some conditions of an aberrant mis-heat treated pipe, a part of or 100% tempered martensite transformed into polygonal ferrite in the structure of the main steam pipe elbow, mainly because of the potential recrystallization provided by the increased internal energy during the bending process^8^. Usually many precipitates in P91 with the pinning effect along prior austenitic grain boundaries (PAGBs) and lath boundaries can inhibit free dislocation movement and subgrain boundary migration, and enhance the creep strength of the normal P91^9^. In the case of the aberrant pipe, the absence of martensitic lath boundaries weakens the effect of dislocation strengthening^7^. At the same time, the absence of martensitic lath boundaries may also result in abnormal growth of the Laves phase. Under operating temperature, the high coarsening rate of the Laves phase leads to the loss of the early positive effect of precipitation-strengthening during the early creep process^10^ and reduces the solid solution strengthening^11^. If the creep life deterioration caused by the aberrant structure directly affects the safe operation of the power unit, and the nuclear or thermal power plant operators will face a safety risk. Therefore, the creep life prediction of critical components, especially those with aberrant mis-heat treated conditions, needs to be carefully assessed for the sake of safe operation of power plants^10,12,13^.

In addition, some literature results have shown that increasing stress^10^, reducing temperature^9^, and prolonging exposure time^14^ may also lead to accelerated cavity growth rates and coalescence, resulting in large-sized cavities and smaller number density of voids. The cavity formation in normal P91 steel is determined by stress, temperature, and exposure time. In this work, a comprehensive analysis is performed to assess the creep fracture mechanism of the aberrant P91 piece.

The in-service aberrant microstructures of the P91 elbow of the main steam pipe that had been in operation for 20,000 h and its creep rupture behavior, hardness variation, and microstructure evolution under a relevant range of temperatures and stresses were investigated in this work. Using the results of the creep experiment, the Larson–Miller constant was accurately determined, and the residual life of aberrant P91 was predicted. Furthermore, the microstructural degradation mechanisms of aberrant P91 heat-resistant steel were also explored.

## Experimental

### Materials

**Figure 1:**
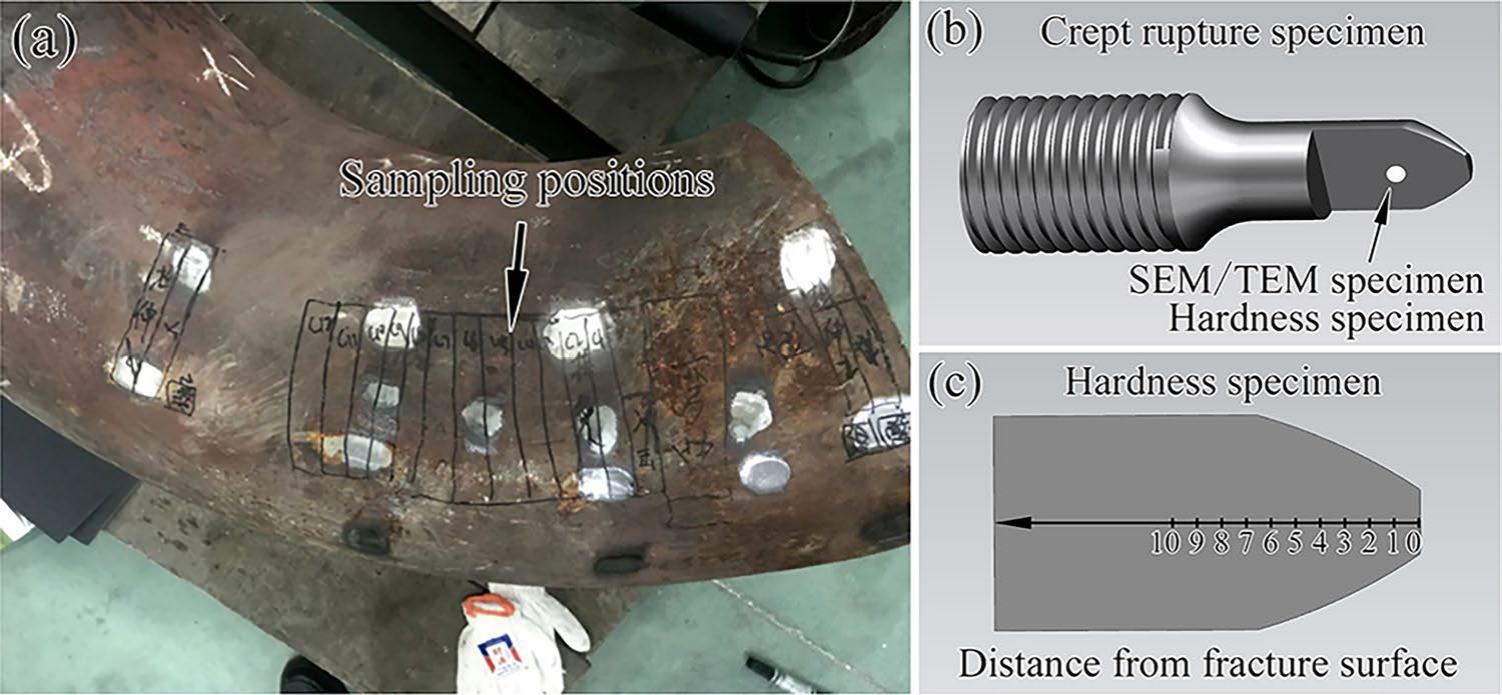
(**a**) Sampling positions of the main steam pipe elbow piece for creep testing, (**b**,**c**) interesting area for microstructure and hardness study.

The initial structure of ASME Gr. 91 pipe steel is a tempered martensite matrix with dense dislocations and precipitates. The P91 piece used in this experiment is a type of aberrant mis-heat treated steel, which has been operated for 20,000 h under around 550 °C and 2.67 MPa of steam. The geometric parameters of the main steam pipe elbow in the power plant are as follows: the outer diameter is 610 mm, and the thickness is 17.45 mm. The chemical composition of the steel is listed in Table 1, and the sampling positions of the main steam pipe elbow piece are illustrated in Fig. 1.

**Table 1:** Composition of the as-received material (wt. %).

Element	C	Si	Mn	Cr	Mo	Ni	V	Nb	Al	Cu	P	S
Content	0.14	0.29	0.45	8.67	0.91	0.17	0.23	0.08	0.005	0.02	0.02	0.002
Code requirement	0.08–0.12	0.2–0.5	0.3–0.6	8.0–9.0	0.85–1.05	≤ 0.4	0.18–0.25	0.08–0.10	≤ 0.04	–	≤ 0.02	≤ 0.01

### Methods

Creep tests were performed in air utilizing a creep machine (RDJ30, Sinotest Equipment Co., Ltd., China). The detailed test parameters are shown in Table 2. The standard stress rupture samples with gauge parameters of Φ10 × 50 mm were processed according to the Chinese national standard GB/T 2039-1997. Micro-hardness values were measured by a Vickers hardness tester (EM1500L, Shanghai Hengyi Precision Instrument Co., Ltd., China) at a load of 50 g (0.49 N) with a dwell time of 10 s. For one material state, five measurements were performed.

**Table 2:** Creep rupture time of the P91steel at different creep temperatures and stresses.

Creep temperature (°C)	Stress (MPa)	Rupture time (h)	Reduction of area (%)
550	120	5524	94.04
	130	1598	94.41
	140	594	92.65
575	100	1550	94.69
	120	363	95.25
	140	54	95.08
600	85	2739	95.33
	95	767	94.49
	105	116	89.46

The microstructural and compositional examinations of samples were observed using a ZEISS SUPRA 35 field emission scanning electron microscopy (SEM) with energy dispersive spectroscopy (EDS). The samples were mechanically ground and polished. The microstructure, distribution, and chemical composition of precipitates of the experimental steels were obtained with a JEOL-2100 transmission electron microscopy (TEM) at an accelerating voltage of 200 kV, and the thin foils were prepared by the ion milling method. The selected area electron diffraction (SAED) was applied to identify the structure of M_23_C_6_, Laves, and MX precipitates. The overall morphology and substructure of the aberrant ferritic matrix were observed by backscattered detection (BSD) using Merlin Compact field emission SEM with EDS technique.

## Result and discussion

### Microstructures the aberrant pipe extracted from service

**Figure 2:**
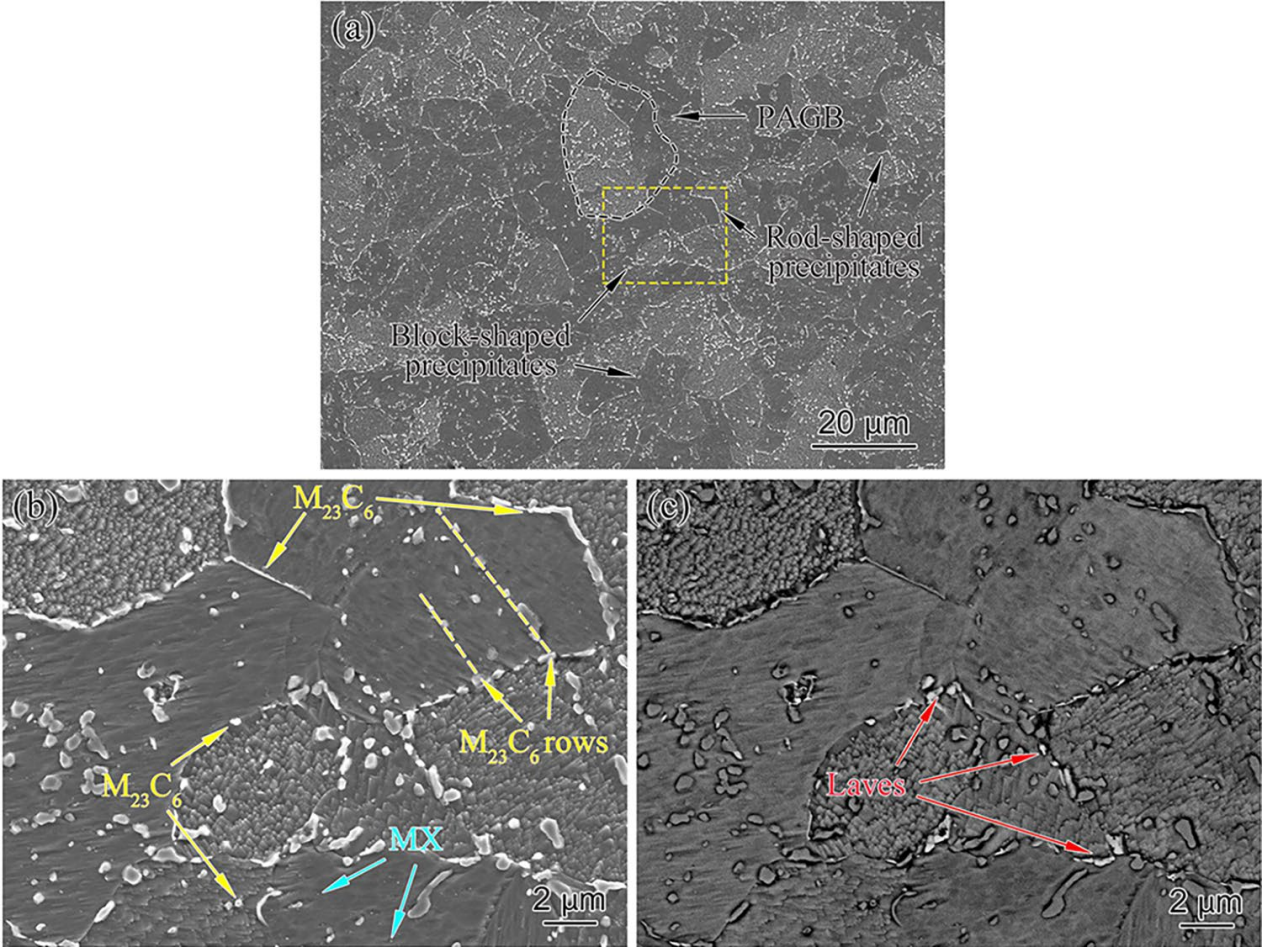
Microstructure of the aberrant P91 steel after service for 20,000 h: (**a**) PAGB morphology and precipitates distribution, (**b**) higher magnification image of the yellow dotted box of (**a**), (**c**) corresponding BSD image of (**b**).

The microstructure of as-received P91 after standard heat treatment contained PAGB, tempered lath and substructures with the dispersion of domain M_23_C_6_ precipitations at various boundaries and grain interior. During short-term service as a superheater and reheater, the significant change is the formation of the Mo-riched Laves phase at PAGB boundaries, while other microstructures are very stable. Figure 2 is the typical SEM micrograph of the aberrant P91 steel operated for 20,000 h. The characteristics of tempered martensite structure (lath blocks) were hard to be seen, which could have decomposed into blocky ferrite and block-shaped or rod-shaped precipitates. This evidence implied that the bend before entering service is mis-heat treated.

The size of PAGB of aberrant heat-resistant steel showed good consistency, ranging from 20 to 30 μm. The precipitates presented in Fig. 2a were amplified and identified by EDS analysis shown in Fig. 2b. The block-shaped or rod-shaped precipitates along the PAGBs, subgrain boundary, and grain interior were the dominant M_23_C_6_, as indicated by yellow arrows. A further observation indicated that the M_23_C_6_ in grain boundaries was coarser than in the grain interior. The diameters of block-shaped and rod-shaped M_23_C_6_ were measured with an average width of 582 nm and length of 1,460 nm, which are significantly bigger than the average size of 248 nm with the as-received one^11^, and that of 481 nm as P91 steel after aging at 650 °C for 20,000 h^15^. The coarsening of M_23_C_6_ after 20,000 h service is supposed to be the mechanism of Ostwald ripening that is the carbon atoms in the steel matrix precipitated as M_23_C_6_ during long service exposure^16^. According to literature reports^4,11^, the finely spherical precipitates could be MX types. The yellow arrows show that some M_23_C_6_ were spread in rows in the grain interior. The row of M_23_C_6_ indicated distribution along the boundaries that could be a trace of the disappearance of the martensitic lath due to the recovery of dislocations within lath boundaries and recrystallization of two boundaries^16^, which proved that the aberrant transition from tempered martensite to ferrite had been completed. Figure 2c shows the BSD images of the Laves phase (marked with red arrows), which give high brightness, because the Laves phase is rich in Mo^17^. Bright Laves particles were located in the vicinity of the boundary with an average size of 637 nm.

**Figure 3:**
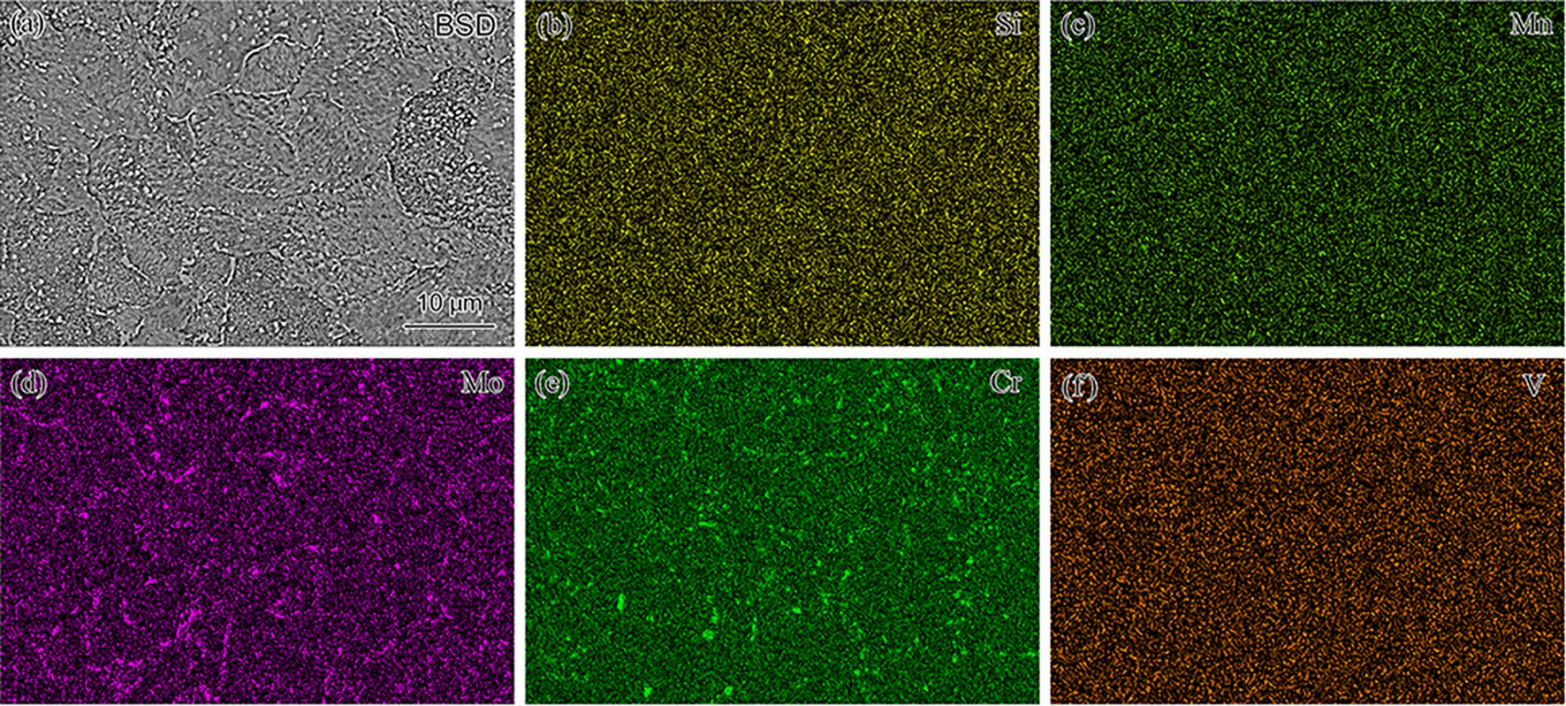
Electron probe microanalysis of aberrant P91 steel operated for 20,000 h.

The elemental mapping of aberrant P91 steel operated for 20,000 h is shown in Fig. 3 showing a uniform distribution of Si, Mn, and V. However, Mo and Cr were inclined to concentrate along the PAGBs and subgrain boundaries due to the formation of Laves phase after long service exposure. The precipitation of the Laves phase devours the M_23_C_6_. The Laves nucleated, grew, and eventually formed a cluster on the PAGBs and subgrain boundaries after creep exposure, consistent with other studies of P91 steels^18^. The leaves phase decreases the content of dissolved Mo in the matrix, which could reduce the solid solution strengthening of P91 steel.

Compared with a new P91 pipe, the martensitic lath of the P91 steel operated for 20,000 h entirely disappeared and transformed into ferrite, and discontinuous precipitated phases occupied some intact grain boundaries. In the normal tempering process of P91 steel, martensite only undergoes recovery transformation. However, the elbow piece was deformed twice during the bending process, which increases a large amount of internal energy to the martensite structure, providing a driving force for the recrystallization of the aberrant ferrite phase^7^. The formation of aberrant ferrite weakens the effect of dislocation strengthening^7^, the Ostwald ripening of M_23_C_6_ weakens the effect of the precipitation strengthening^19^, and the Laves phase reduces the solid solution strengthening^11^. The three superposition effects significantly influence the strength, plastic toughness, and high-temperature creep strength of P91 steel in this study.

### Creep rupture behavior

**Figure 4:**
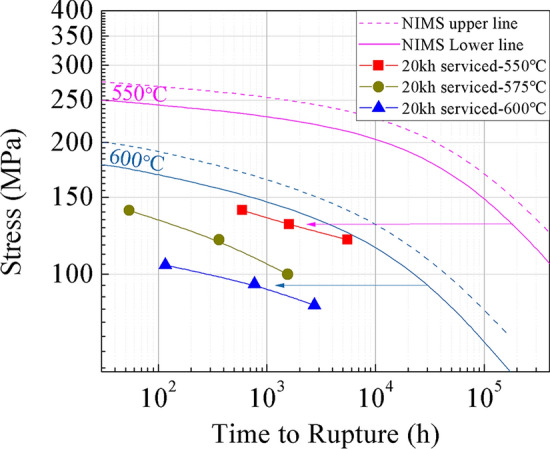
Comparison of creep rupture strength of the aberrant P91 steel after service for 20,000 h with the NIMS creep data sheet^20^.

Figure 4 shows the creep rupture strength of the aberrant P91 steel, which was compared with the National Institute for Materials Science (NIMS) creep data sheet on the received one^20^. In practice, the Grade 91 steels before use should have a creep strength better than the lower line, as shown in Fig. 4. However, the creep rupture life of the aberrant P91 piece after service for 20,000 h is reduced by nearly two magnitudes at the test temperatures.

**Figure 5:**
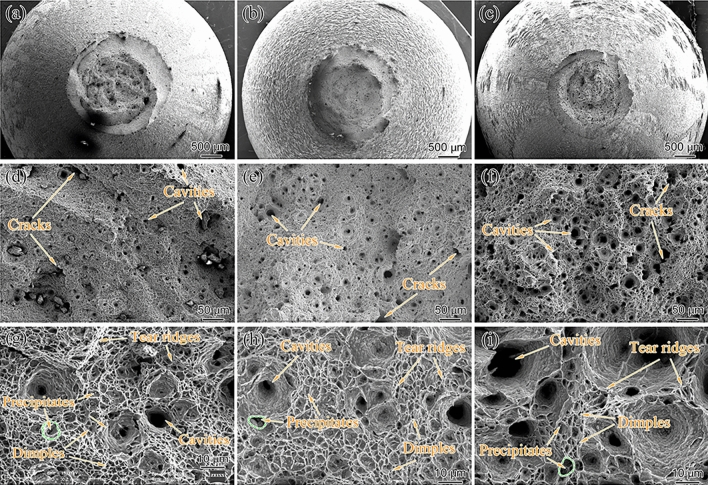
Fracture surface morphology for the creep ruptured specimens at: (**a**,**d**,**g**) 550 °C/120 MPa, t_r_ = 5524 h; (**b**, **e**, **h**) 575 °C/100 MPa, t_r_ = 1550 h; (c,f,i) 600 °C/85 MPa, t_r_ = 2739 h.

The effects of creep temperature and stress on the rupture time of aberrant P91 steel are listed in Table 2. Specimens under the different creep temperatures from low to high stress all presented dimple-mode ductile fracture, so only the fracture surface of the samples with the lowest stress under the corresponding creep temperature is given in Fig. 5. A lot of circular dimples and cavities existed in the micrography of the fracture surface for aberrant P91 samples at lower magnification. Besides, the dimples, cavities, cracks, and tear ridges can be revealed at higher magnification. As can be seen from Fig. 5d,g, many cavities and dimples, with a high density of dimples dispersed around the cavities observed under 550 °C. With an increase in creep temperature up to 575 °C, the number and density of dimples began to decrease while that of cavities increased significantly; simultaneously, the size and depth of the cavities increased considerably, as shown in Fig. 5e,h. When the temperature eventually increased to 600 °C, the size of cavities and depth grew dramatically, and the proportion of cavities in the entire visual field reached a relatively high degree, which is presented in Fig. 5f,i.

The reduction of area after fracture of the creep rupture samples with increasing temperature was 94.04, 94.69, and 95.33%, respectively, indicating severe plastic deformation in the near fracture region at 600 °C for 85 MPa. The percentage reduction of area after fracture was consistent with those of the microscopic microstructure. Green circles in Fig. 5g,h,i demonstrate the cavity formation around a precipitate, which illustrates that the precipitates promote the formation of cavities. Small cavities grow during the creep and eventually merge into big cavities, leading to fractures. Our study of aberrant P91 steel indicates that the cavity size produced under low temperature and high stress conditions is smaller than that under high temperature and low stress conditions. However, other works of literature have demonstrated that increasing stress^10^, reducing temperature^9^, and exposure time^14^ also result in greater cavity growth rates and coalescence, which leads to larger size and smaller number density of cavities. Stress, temperature, and exposure time directly determine the cavity formation for normal P91 steel. Therefore, a comprehensive analysis is needed to grasp the creep fracture mechanism of the aberrant P91 steel.

**Figure 6:**
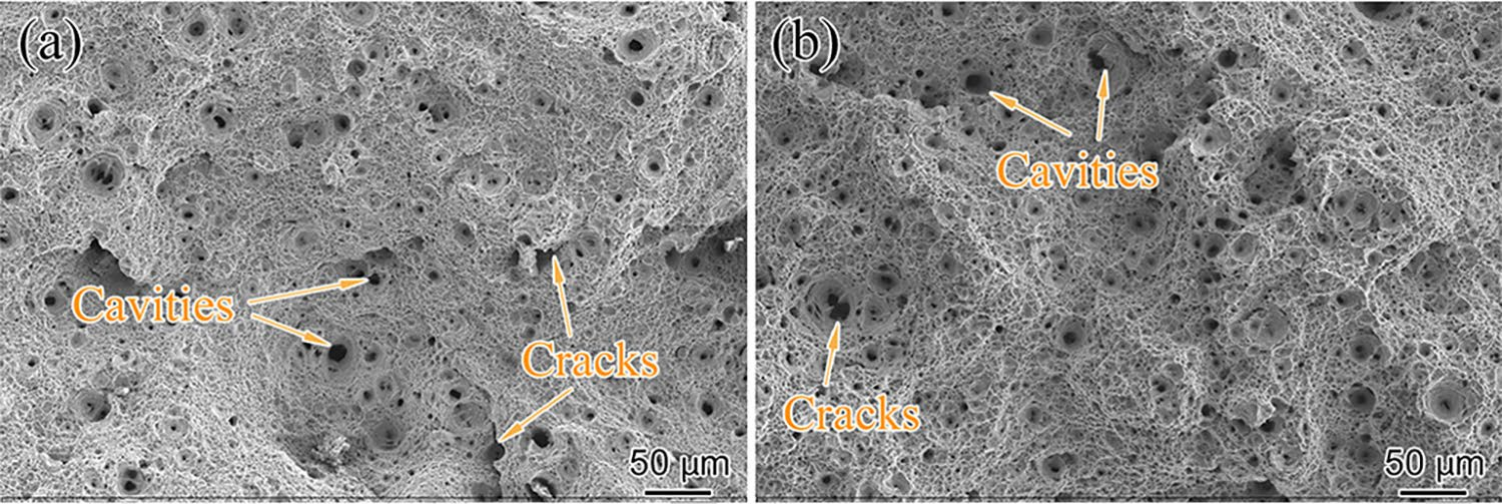
Fracture surface at high magnification for creep ruptured samples with (**a**) 550 °C/140 MPa, t_r_ = 594 h, (b) 575 °C/140 MPa, t_r_ = 54 h.

Figure 6 illustrates the rupture surface of the specimens under 140 MPa at different temperatures. By comparing Fig. 6a and Fig. 6b, it can be seen that with the increase in creep temperature from 550 to 575 °C, the density and size of cavity increased. However, the rupture time of the specimen under 575 °C only operated for 54 h. The above analysis indicated that under the same stress, the process of cavity growth is more sensitive to the increase in temperature than the extension of exposure time. Furthermore, combined with the fractograph morphology, we found that under the same creep temperature, the cavity size increased as the exposure time extended (i.e., the smaller the stress). The growth of the cavity is sensitive to the extension of exposure time under the condition of constant temperature. We can infer that for the aberrant P91 piece in this study, the influence of temperature on cavity size is crucial, followed by the effect of stress.

**Figure 7:**
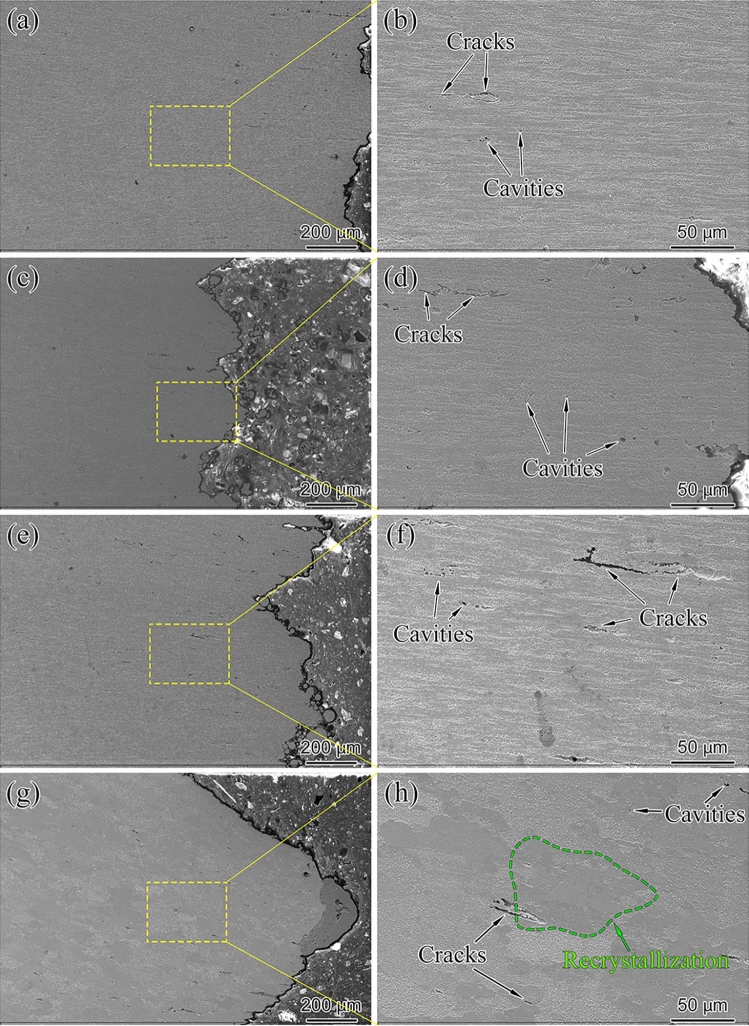
Fracture cross-section characteristic for the creep ruptured specimens tested at: (**a**,**b**) 550 °C/130 MPa, t_r_ = 1598 h, (**c**,**d**) 575 °C/100 MPa, t_r_ = 1550 h, (**e**,**f**) 600 °C/95 MPa, t_r_ = 767 h, (g,h) 600 °C/105 MPa, t_r_ = 116 h.

The formation of cavities and initiation of cracks in fracture cross-section are shown in Fig. 7. Different from the intergranular crack propagation, the cavity connection under the action of axial stress formed the crack. Fig. 7a–d compared the effects of temperature on cavity size and cracked width under almost the same rupture time. With the creep temperature increased from 550 to 575 °C, the cavity size and crack width exhibited a noticeable increase, indicating that the higher temperature has a significant effect on the microstructure evolution of the diffusion-controlled. After being tested at 600 °C as shown in Fig. 7e,f, the cavity size and crack width were the largest, although the stress was reduced compared with the specimens tested at 550 and 575 °C, which also confirms that the influence of temperature on the cavity and crack is greater than the stress for the aberrant P91 steel.

### Hardness variation

**Figure 8:**
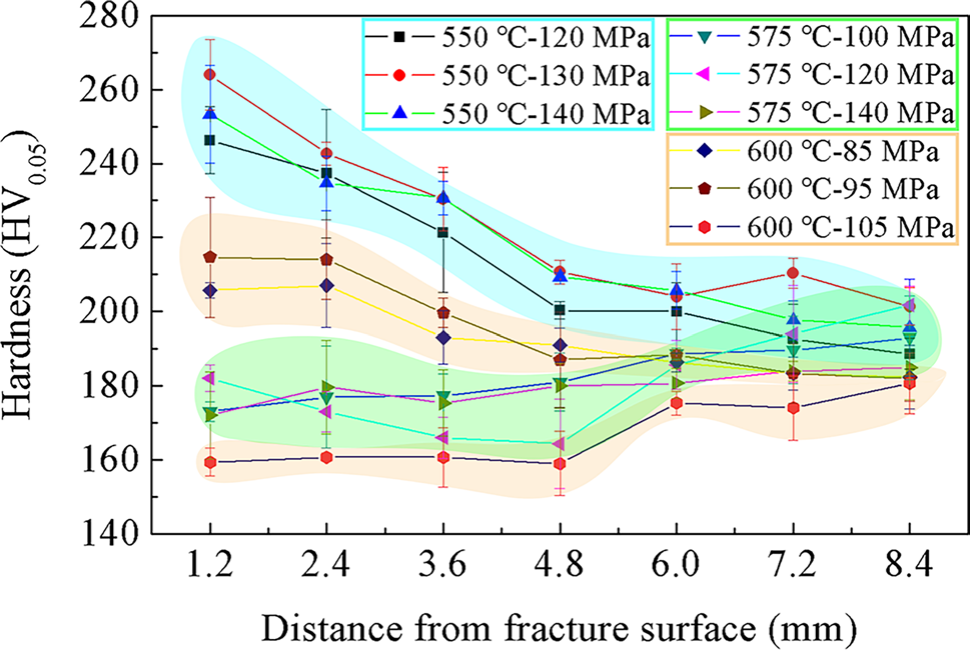
Hardness distribution for creep rupture failed specimens.

Figure 8 shows the variation of micro-hardness HV_0.05_ with the position on the gauge of the creep rupture specimens. The gauge portion of the aberrant creep rupture specimen exhibited various hardness trends according to different exposure temperatures. The hardness trend of the samples at 550 °C (for all stress) and 600 °C (for 85 and 95 MPa) decreased with distance from the fracture site. However, close to the fracture surface, a slight drop in the hardness can be observed compared to that of the rest of the gauge portion of specimens at 575 °C (for all stress) and 600 °C (for 105 MPa). The difference in hardness variation trend may be related to the creep damage evolution mechanism. Figure 7e,f and (g,h) exhibit completely different microstructures under creep conditions of 600 °C, 95 and 105 MPa. The higher stress (105 MPa) resulted in significant recrystallization, and elongated grains transformed into equiaxed grains. In addition, by comparing Fig. 7a–d, it can also be found that compared with 550 °C, the shape of elongated grains at gauge portion under 575℃ creep condition has not changed significantly, but the grain boundaries have become blurred. The morphology transformation suggests that the sample is already in the recrystallization stage under the creep exposure condition of 575 °C, but the structure has not been completely reconstructed into new equiaxed grains. The present results show that these microstructural transformations and softening processes, i.e., the result of recrystallization, both operate under creep conditions with high temperature and high stress. Due to the interaction of the work hardening and recrystallization at the necking of the gauge portion, the hardness distribution of the sample shows two different trends.

The average hardness of aberrant P91 steel operated for 20,000 h was 175.2 ± 6.8 HV_0.05_. This study also shows a slight variation in hardness between the gauge far away from the fracture surface and the grip portion, which is consistent with other research findings^7,21^. As mentioned above, the matrix structure of the aberrant P91 piece operated for 20,000 h was transformed into ferrite, weakening dislocation, precipitation, and solid solution strengthening capabilities. Hence, the hardness of aberrant P91 was only 175.2 ± 6.8 HV_0.05_, which is less than 200–250 HV_0.5_ for normal P91^7^ and does not meet the hardness standard of BPVC. CC. BPV in ASME for P91 (185-248HBW). Recrystallization under high temperatures and stress conditions causes the loss of subgrain boundaries as a major obstacle against dislocation motion, weakening dislocation strengthening and further reducing the hardness of aberrant P91^15,22^. The structure evolution accelerates the softening of hardness, resulting in premature creep failure of in-service steel^23^. Hence, non-destructive measurements utilizing hardness is a solid foundation for evaluating creep life.

### Microstructure evolution after creep testing

**Figure 9:**
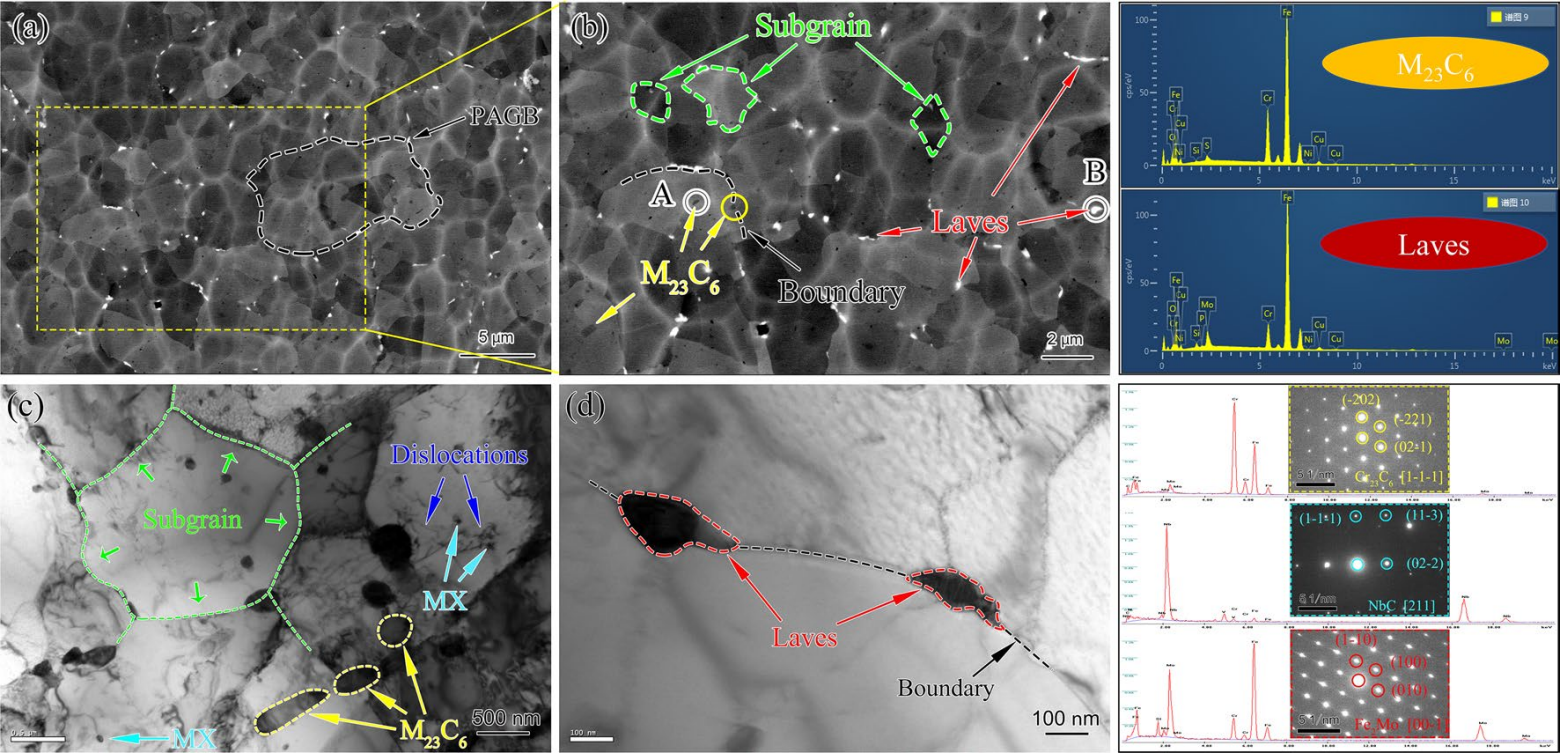
Microstructure of the creep ruptured specimen at 550°C for 140 MPa (tr = 594 h): (**a**) aberrant ferritic matrix observed by BSD, (**b**) higher magnification image of (**a**), (**c**,**d**) typical structure of subgrain, dislocation, M_23_C_6_, MX and Laves observed by TEM; corresponding EDS and SAED are indicated in the right insets.

Figure 9 shows the microstructure of the specimen after creep failure at 550 °C for 140 MPa (t_r_ = 594 h). The PAGBs and absence of lath boundaries can be clearly seen in Fig. 9a,b from BSD images. Noted that the white mesh stripes were the damage marks produced by the process of ion milling of the TEM specimen. The Cr-rich M_23_C_6_ were block-like or rod-like elongated precipitates distributed along the PAGB or in the grain, while the Mo-rich Laves phase mainly appeared in the PAGBs. The subgrain can be seen in the gauge portion of aberrant ferritic steel, as revealed in Fig. 9b, and the coarsening trend of the subgrain can also be seen, as shown in the green arrow in Fig. 9c. The M_23_C_6_ distributed along the subgrain boundaries can stabilize subgrains by applying pinning forces that impede the recrystallization^22^. The dislocation networks constitute the subgrain boundaries, which are the hard region in the microstructure of ferritic steel and can be expected to provide high creep strength^24^. The Nb-rich MX is present mostly inside the grains, the same as another research result^4^, possibly because fewer subgrain boundaries are left in the aberrant ferritic. Figure. 9d revealed that the Laves phase eventually gathers a cluster on PAGBs after a specific creep operation. The high hardness of the Laves phase is also a reason for the formation of the creep cavity^11^. In addition, the precipitation of the undesirable Laves phase causes loss of solid solution strengthening and accelerates the subgrain coarsening, leading to decreased creep properties^23^.

**Figure 10:**
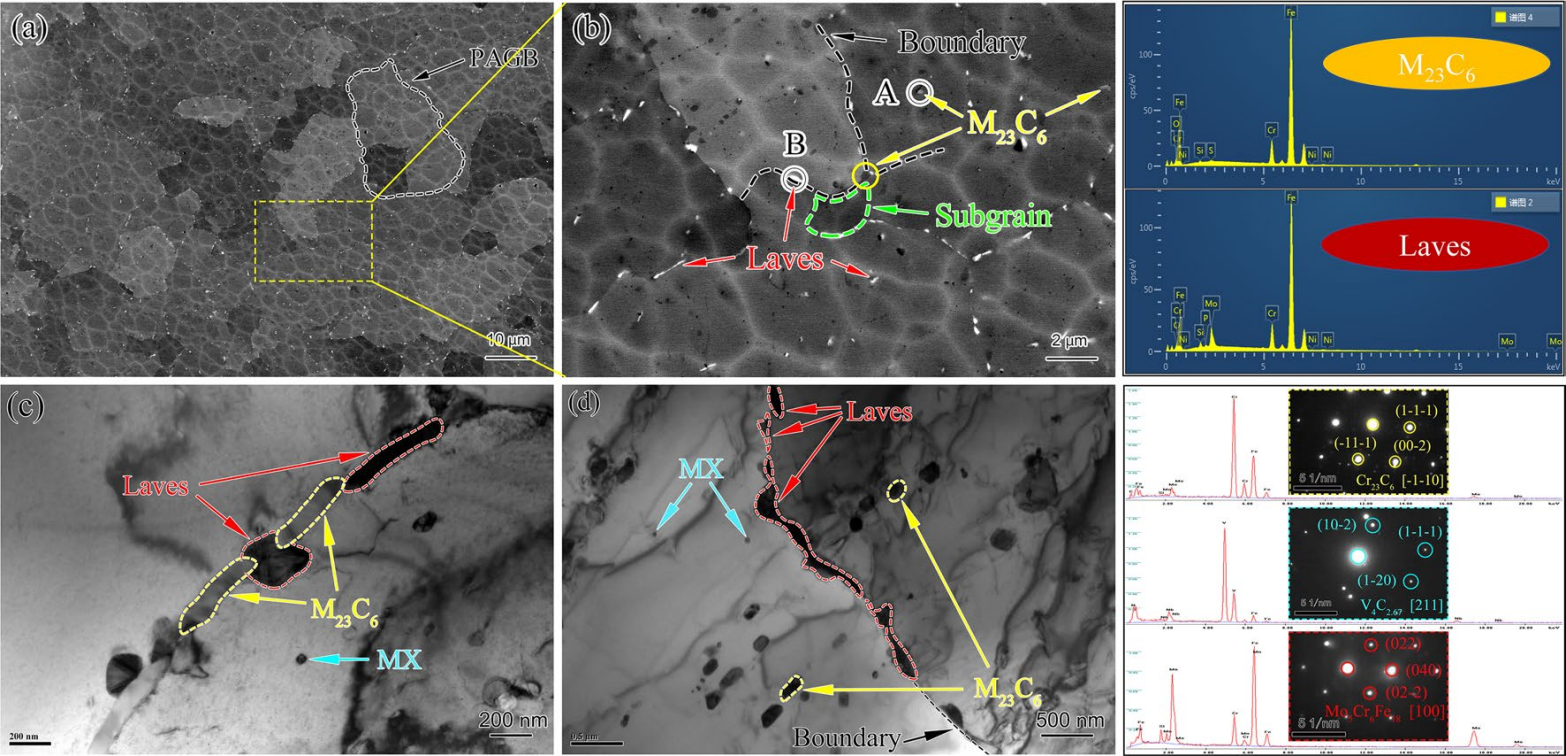
Typical microstructure of the creep ruptured specimens at 575°C/100 MPa (tr = 1550 h): (**a**) aberrant ferritic matrix observed by BSD, (**b**) higher magnification image of (**a**), (**c**,**d**) typical structure of subgrain, M_23_C_6_, MX and Laves phase observed by TEM. The corresponding EDS and SAED are indicated in the right insets.

Figure 10 shows the typical BSD and TEM images of the creep ruptured specimen at 575 °C/100 MPa/ (tr = 1,550 h). Comparing with Fig. 9a,b and Fig. 10a,b, it can be observed that the significantly decreased subgrain density with the coarsening of the subgrain is attributed to the migration and unknitting of dislocation^25^. Subgrain boundaries, in turn, constitute a significant obstacle to dislocation movement^15^. The decrease of the subgrain density further leads to the reduction in hardness, as can be seen from the hardness data away from the fracture surface in Fig. 8, which agrees with Armaki’s results^22^. In addition, the 575 °C creep ruptured specimen shows a decrease in dislocation density due to dislocation annihilation and rearrangement, and there is no interaction area between MX and dislocation, as shown by the blue and teal blue arrows in Fig. 9c. It is found that the spacing of MX precipitated phase did not change significantly with creep temperature increasing from 550 to 575 °C, which is consistent with the literature pointing out that MX has high thermal stability^23^. Corresponding to no deterioration of the performance of the Z-phase was found. Figure 10c certifies that high-temperature creep promotes the precipitation of the Laves phase at PAGBs and forms a cluster around the M_23_C_6_ after a certain creep time. Figure 10d further confirms that the Laves phase cluster along the PAGBs with a large area, and the linear size within the visual range was close to 3.4 μm. The precipitates preferentially nucleate at PAGBs, lath boundaries, and subgrain boundaries^10,11^. Due to the loss of the lath boundary and the decrease of subgrain boundaries for aberrant P91 steel in this study, a cluster of Laves phase precipitates at PAGBs. The formation of large Laves has a severe impact, which causes PAGBs to break away from the pinning effect of the precipitates and significantly weakens the solid solution strengthening^11^. Therefore, forming the large area Laves phase limits the life of aberrant heat-resistant steel.

### Creep life prediction

**Figure 11:**
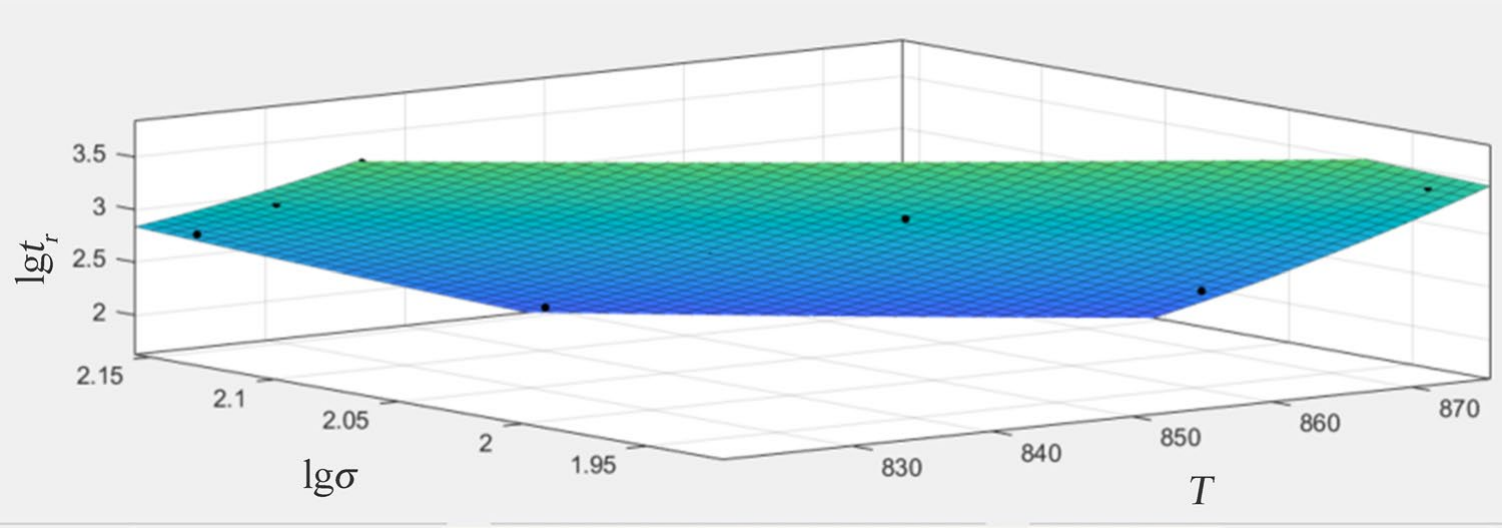
Larson-Miller parameter fitting results.

Larson–Miller method (LMP) is an effective way to assess the effects of operating temperature and service time on creep behavior^10,26^, which is given by Eq. (1).

1 $$LMP = T\cdot\left( {C + \lg t_{r} } \right)\,$$

where *T* is the temperature in degrees Kelvin, *t*_*r*_ is the rupture time, and C is constant, which approximately equal to 20 for most steel^25^. Some experimental data have proved that the values of C mainly rely on carbon content^27^, and the optimal ranges of the values are narrow^6,21,28,29^. Since predicting the remaining life involves exponential changes of time with the parameters, the deviation of each parameter leads to serious prediction errors, which means that the value of C needs to be accurately calculated. MATLAB code has been applied to calibrate the material constants of twelve models such as Larson–Miller, Manson–Haferd, Manson–Brown, Orr-Sherby-Dorn and so on, and proved to have good accuracy^30^. Other studies have shown that the quadratic polynomials is sufficient and that the use of higher degree polynomials is superfluous^31^. To obtain an accurate value of the Larson-Miller constant, we expressed LMP equations as quadratic polynomials^32,33^ based on MATLAB. LMP, T (C + lgtr) and lgt_r_ can be described by:

2 $$LMP{\text{ = a}}_{{\text{0}}} {\text{ + a}}_{{\text{1}}} {\text{(lg}}\sigma {\text{) + a}}_{{\text{2}}} {\text{(lg}}\sigma {\text{)}}^{{\text{2}}}$$

3 $$T\cdot(C + \lg t_{r} ) = a_{0} + a_{1} (\lg \sigma ) + a_{2} (\lg \sigma )^{2}$$

4 $$\lg t_{r} = \frac{{a_{0} + a_{1} (\lg \sigma ) + a_{2} (\lg \sigma )^{2} }}{T} - C$$

where *σ* is the stress, and a_0_, a_1_ and a_2_ is the coefficient of the polynomial, respectively. Taking the creep experimental data into Eq. (4), the calculated curve fitting with a confidence level of 95% using the MATLAB curve fitting toolbox. The fitting results and relevant data are shown in Fig. 11 and Table 3, respectively. According to the fitting analysis, when the value of C was 33.65, and the value of a_0_, a_1_, and a_2_ was 9.53 × 10^4^, − 5.23 × 10^4^, and 1.02 × 10^4^, respectively. Shrestha et al.^21^ have reported that the typical empirical value of C is 33 for the P91 steel, which is almost the same as the result obtained by the MATLAB method for the aberrant piece in this study. The best correlation coefficient (R2) calculation reaches 0.97, which was close to 1, indicating the accuracy of the fitting result.

**Table 3:** Data obtained by MATLAB fitting.

C	a_0_	a_1_	a_2_	SSE	R^2^
23.65	− 2.95 × 10^4^	− 1.77 × 10^5^	− 1.90 × 10^4^	–	–
43.66	2.20 × 10^5^	6.75 × 10^4^	3.94 × 10^4^	–	–
33.65	9.53 × 10^4^	− 5.23 × 10^4^	1.02 × 10^4^	0.16	0.97

According to Eq. (5), the allowable stress (σ_a_) of the main steam pipe with an operating pressure of 2.67 MPa in service is 70 MPa.

5 $$\sigma_{a} = \frac{nPD}{{2\delta }}$$

**Figure 12:**
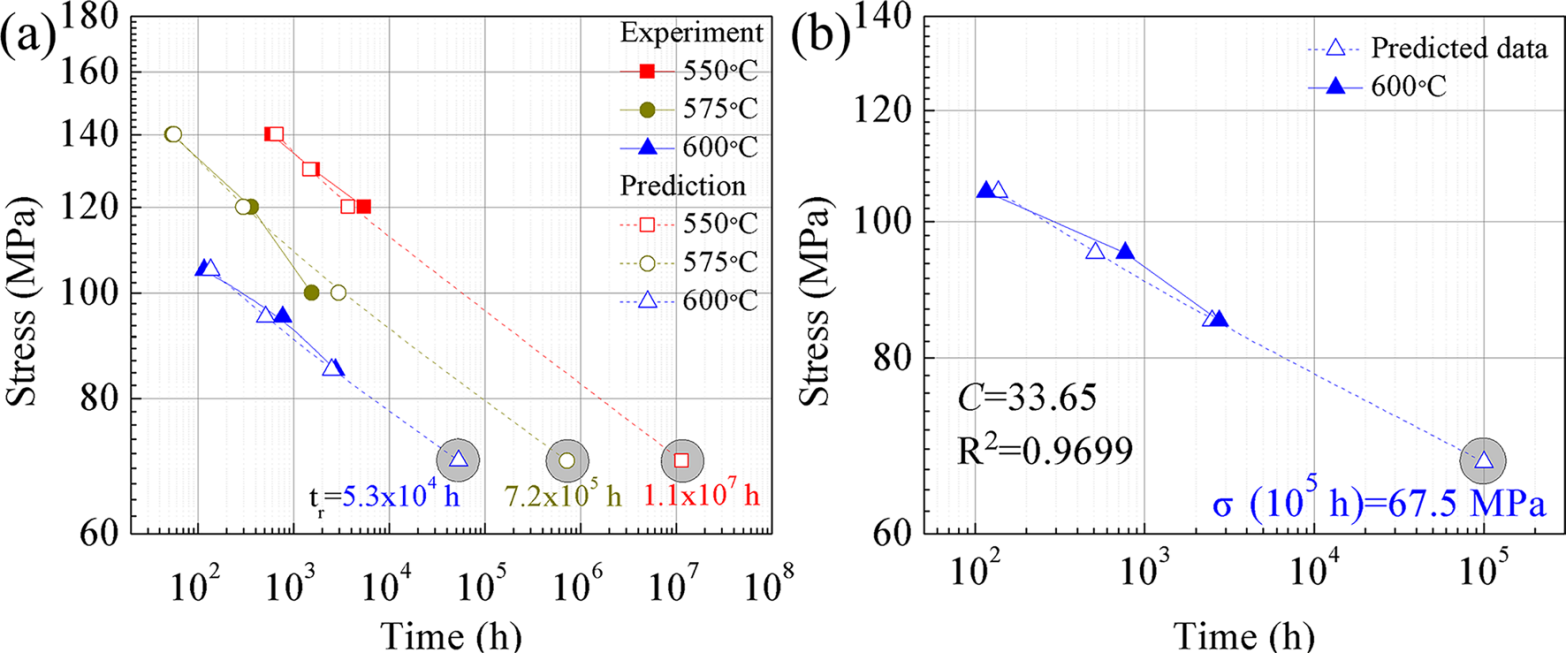
Larson-Miller plot of creep rupture data for P91:(**a**) allowable stress rupture life prediction compared with experimental data, (**b**) extrapolating stress for a rupture life of 100,000 h under 600 °C

P, D, δ, and n are the main steam pipe elbow's operating pressure, diameter, wall thickness, and safety coefficient. The safety factor coefficient is generally chosen as 1.5. Taking the various operating temperatures and allowable stress into Eq. (4), the prediction curves of the stress rupture life of P91 heat-resistant steels were calculated using the LMP method, presented in Fig. 12a. At the temperatures of 550, 575, and 600 °C, creep life increases with the lowering of the applied stress. The fitted plots were basically consistent with the creep testing results, showing sound-fitting effects on the experimental data. The residual life time of P91 performed at 550, 575, and 600 °C was 11,447,201, 722,039, and 53,353 h, respectively. The main steam pipe elbow is in service at about 550 °C. The creep test conditions are too ideal and can not fully simulate the actual working state of P91 steel. Therefore, the ambient temperature in the assessment parameter is selected as 600°C to obtain the extrapolating life^34^. With 20,000 h of service under pre-test conditions, the service life of aberrant P91 steel under operating conditions can reach more than 73,353 h. There is still a large gap between the service life and the 100,000 h plant operators expect^35^. The service life of the aberrant microstructure can not guarantee the safety operation to the greatest extent of the main steam pipe elbow.

The extrapolating strength for the main steam pipe elbow with aberrant microstructure from tests up to 5524 h at 600 °C was analyzed to predict by the time–temperature parameters (LMP), seen in Fig. 12b. The extrapolating strength under 600 °C for P91 steel predicted by the LMP method and the data from other works of literature^10,21,34,36–38^ are listed in Table 4. Based on a large scale of creep tests in the temperature range of 600–700 °C and at stresses 35–350 MPa, the extrapolating 100,000 h stress of Grade 91 CL2 steel of 91 MPa for 600 °C is determined using the LMP method^21^. The extrapolating stress range of 100,000 h creep rupture stress at 600 °C for P91 steel in Table 3 is between 83 and 92 MPa. A new assessment of creep rupture stress for Grade 91 steel is based on the European Creep Collaborative Committee (ECCC) data sheet in 1995 that had passed 100,000 h in the range 575–625 °C, which calculated the 100,000 h value at 600 °C is 90 MPa, according to the LMP method^34^. According to the last ECCC data sheet in 2019^39^, the 100,000 h value at 600°C is 84.2 MPa. The long-term creep result exhibited that the rupture stress of P91 steel for 112,431 h at 600 °C is 80 MPa^36^, which means the allowable stress under 100,000 h must be greater than 80 MPa. The extrapolating strength of the LMP method is in good agreement with ECCC creep data and experimental data. Moreover, the extrapolating 100,000 h stress of 67.5 MPa by LMP for aberrant P91 steel in this study, which is lower than the predicted values from other works of literature, indicating aberrant microstructure exhibits reduced creep resistance, and the safety of the main steam pipe elbow must be further evaluated in the later service period.

**Table 4:** Extrapolating stress for rupture time as long as 100,000 h of P91 under 600 °C by LMP method and experimental data.

Method	Extrapolating stress (MPa)	Reference
LMP	91	Shrestha et al.^21^
LMP	92	Srinivasan et al.^38^
LMP	83	Wilshire et al.^37^
ECCC^a^ + LMP	90	Bendick et al.^34^
ECCC^b^	84.2	ECCC data sheet ^39^
Experiment (113,431)	80	Panait et al.^36^
LMP	67.5	This study

^a^ECCC data sheet in 1995.

^b^ECCC data sheet in 2019.

## Conclusions

In this work, the creep behaviors of the aberrant P91 main steam pipe elbow after service for 20,000 h were performed in the temperature range of 550–600 °C and at stresses 85–140 MPa. The results are summarized as follows:

1. The martensitic lath of the P91 steel operated for 20,000 h entirely disappeared and transformed into the aberrant ferrite. Discontinuous precipitated replaced some intact grain boundaries. The recrystallization of the elbow decreased the hardness of the aberrant P91 to 175.2 ± 6.8 HV_0.05_, which is less than 200–250 HV_0.5_ for normal P91 and does not meet the hardness standard of BPVC. CC. BPV in ASME for P91 (185-248HBW).
2. The creep results (i.e., 550 ℃ and 575 ℃ test comparison) showed that the stress and rupture life reduced, but the cavity size and crack width were the largest, which confirms that the influence of temperature on the cavity and crack is more significant than that of the stress for the aberrant P91 steel.
3. The high stress and temperature resulted in significant recrystallization, and elongated grains were transformed into equiaxed grains. Due to the interaction of the work hardening and recrystallization at the necking of the gauge portion, the hardness distribution of the sample shows two different trends.
4. After creeping till failure at 575 °C for 100 MPa, due to the loss of the lath boundary and the decreased of subgrain boundaries for aberrant P91 steel, large clusters of Laves phase with an aggregation size approaching 3.4 μm precipitated at PAGBs, which causes PAGBs to break away from the pinning effect of the precipitates and significantly weakens the solid solution strengthening.
5. According to the extrapolation of the Larson–Miller model, the service life of aberrant P91 steel under operating conditions can reach more than 72,549 h, which is short of the 100,000 h of service life expected by factory operators.

## Data Availability

The datasets used and analyzed during the current study are available from the corresponding author on reasonable request.
